# Developing a Culture to Facilitate Research Capacity Building for Clinical Nurse Consultants in Generalist Paediatric Practice

**DOI:** 10.1155/2013/709025

**Published:** 2013-07-16

**Authors:** Lesley Wilkes, Joanne Cummings, Nicola McKay

**Affiliations:** ^1^Clinical Nursing Research Unit, Family and Community Health Research Group (FaCH), School of Nursing and Midwifery, University of Western Sydney, (UWS) and Conjoint Appointment with Nepean Blue Mountains Local Health District (NBMLHD), Australia; ^2^Clinical Nursing Research Unit, Nepean Hospital, P.O. Box 63, Penrith, NSW 2751, Australia; ^3^Clinical Nursing Research Unit, Nepean Hospital, School of Nursing and Midwifery/UWS & NBMLHD, P.O. Box 63, Penrith, NSW 2751, Australia; ^4^Paediatric Clinical Nurse Consultant, Western Sydney Local Health District/NSW Child Heath Network, Paediatric Unit Mt Druitt Hospital, Australia

## Abstract

This paper reports a research capacity building exercise with a group of CNCs practicing in the speciality of paediatrics in New South Wales (NSW), Australia. It explores the first step in building a research culture, through identifying the research priorities of members of the NSW Child Health Networks Paediatric Clinical Nurse Consultant group, and this forms the major focus of this paper. A nominal group technique (NGT) was utilised with sixteen members to identify research topics for investigation which were considered a priority for improving children's health care. The group reviewed and prioritised 43 research topics in children's health which were identified in the literature. As a result of conducting this research prioritisation exercise, the group chose two research topics to investigate: reasons for children representing to the Emergency Department and a comparison of the use of high-flow and low-flow nasal prongs in children with bronchiolitis. The research team will continue to mentor the nurses throughout their research projects which resulted from the NGT. One bridge to leadership development in enhancing patient care is translating knowledge to practice and policy development. This study leads the way for a group of CNCs in paediatric nursing to combine their research capacity and influence clinical knowledge.

## 1. Introduction

The clinical nurse consultant (CNC) role was established in New South Wales (NSW), Australia, in the late 1980s [1, 2]. It is equivalent to the clinical nurse specialist role in the United States of America [1, 3] and United Kingdom [4]. Heals [5] states that part of the CNCs role is to improve clinical practice, facilitate change, disseminate evidence-based practice, and improve communication in and beyond the health team.

There has been continued confusion about the CNC role [1, 2, 6]. In 2005, NSW Health reaffirmed the five domains of practice for CNCs in NSW as clinical service and consultancy, clinical leadership, research, education, and clinical service planning and management [7]. CNCs are required to dedicate their time evenly to each of these domains, however, a study by O'Baugh et al. [1] found that within the research domain less than 60% of CNCs surveyed played a significant part in the development of clinical research. This trend was confirmed in a study in Victoria, Australia, by Bloomer and Cross [6] where a similarly titled role existed. In both studies, the CNCs cited lack of support and workload as reasons for not being able to enact all domains, (in particular, leadership and research), as health service management tends to place a greater emphasis on the clinical service and consultancy domains [2]. This paper reports on a team and mentoring approach which was implemented by the authors, in 2012, to assist a group of generalist paediatric CNCs practising in NSW to comply with the demands of the research domain. The first step in this research capacity building was to determine the research priorities of the group. 

## 2. Background

A nursing research culture “may involve an organisation constructing an environment that enables and supports creative work to generate new knowledge that provides researchers with opportunities to interact and grow” [8]. An enabling research culture is essential to building research capacity (ability to plan and conduct research) in nursing. This research culture is characterised by research productivity, positive collegial relationships, inclusiveness, noncompetiveness, and effective research processes and training [8]. Nurses need to develop their research capacity, that is their ability (or skill level) to undertake research projects [8–11]. The fundamental motivation for this capacity building is to optimise research performance with high-quality outcomes in the academic and clinical arenas [9–11]. An enabling research culture has, at its centre positive collegial relationships, productivity, inclusiveness, and effective research processes and training [8, 12, 13]. One step in establishing collegial relationships with common goals is for a group to determine their research priorities. The setting of research priorities is an established way to provide research direction for health services and groups [14, 15]. This may aid the development of cross-organisational and professional collaboration (depending on who the members of the group are) and ultimately may aid in establishing research culture [16]. Further, setting research priorities allows nurses to explore important issues in an era when the research dollar is shrinking [17, 18] and enables a direct link between nursing research and the development of healthcare practice [16]. 

When setting research priorities not only do nurses take an interest in future research, but also their commitment will initiate knowledge development within clinical practice [16] and hopefully fuel an enabling research culture among clinicians. Another essential element for developing a research culture and producing research outputs is developing the capacity of the nurses who need to conduct the research [19, 20]. This capacity building can be initiated by the nurses becoming informed and keen consumers of research [21]. Unless nurses' research capacity is directed and translated by nurse specialists such as CNCs to build patient care, there will be a significant gap between knowledge generation and usage [22]. This aspect of building research capacity will assist nurses to implement their research findings. Systematic reviews of the literature are an essential part of this development so that policy is based on strong evidence [23, 24]. 

Developing research capacity can be accomplished in a number of ways including formal education through postgraduate degrees in nursing, in-house informal research education programs, and involvement with successful research teams [12, 25, 26]. Mentoring is considered to be an essential aspect of building the research capacity of clinical nurses [27, 28]. This mentoring involves a senior experienced professional working with less advanced nurses to aid them in developing research skills and knowledge. This mentoring must be sustained and the group motivated to be successful [29]. Byrne and Keefe [27] conducted a literature review of Medline and CINAL databases between 1990 and 2001, and they found that a mentor working with a group of nurses to set research priorities and objectives for a given nursing setting was an optimal method to produce scholarly research. 

Research priority setting in paediatric nursing, including specialties, has been a focus of studies since the 1990s [15, 30–33]. In America, a Delphi Technique has been used by both Schmidt et al., [31] and Broome et al. [30] to determine the research priorities for paediatric nurses. The Schmidt study identified the top five priorities as analgesic drip weaning, central line dressings, analgesic dosing, procedural pain, growth and development knowledge [31]. The Broome et al. study identified their top six priorities as interventions to prevent repeated child abuse, efficacy and quality of paediatric home care, postoperative pain relief, educating for effective parenting skills, and strategies to manage pain in infants [30]. 

Wilson et al. [15] conducted a study in Western Australia in 2005-2006 using a randomised selection of registered nurses at a paediatric referral hospital. Using a Delphi Technique their top five priorities were reducing medication errors, impact of pain assessment on pain management, exploring new health promotion strategies, impact on family and support required for children needing long-term care, school and antenatal health education programs for children and parents, and reasons for parent noncompliance with treatment. 

As part of a research mentoring program for paediatric CNCs across NSW, it was decided that the group should determine their research priorities and establish a relationship with the team. The CNCs felt that through conducting clinical research they would raise their profile in the paediatric health community and in so doing, improve child and adolescent health care in the state and internationally. This paper focuses on the process of establishing research priorities for the group and, in so doing developing a research relationship with each other and a mentor, who was a member of the team.

## 3. Method

### 3.1. Research Design

The use of consensus methods, such as Delphi surveys or nominal group techniques (NGT), is common when developing research priorities to guide the commissioning of health research [15–17, 30, 31, 34, 35]. This study utilised a quantitative NGT [36–38] with a group of paediatric CNCs. An NGT provides an orderly procedure for obtaining relevant and reliable information from a group of experts within a focus group setting or a small group meeting [39]. NGT is a method which promotes creative and meaningful interpersonal disclosures from the participants by gathering equally weighted responses that can offer valid representations of the group's views [40]. The collaborative nature of NGT increases the likelihood that the group will work together on problem identification, generate research questions, and develop solutions to change and enhance nursing practice and policy [39]. The monthly meetings held by the CNCs provided a forum in order to conduct the NGT. This made the process efficient and effective with immediate feedback to the group. 

### 3.2. Setting

The study was conducted in a meeting room of the Neonatal Emergency Transport Service (NETS) in Sydney, NSW, Australia. NSW has the largest populace in Australia. In 2011 the population of children under 15 years of age was 1.36 million [41].

### 3.3. Study Population

In the NSW public health service and within the structure of the NSW Child Health Networks (CHN) there are a total of 23 CNCs providing generalist paediatric nursing care, across urban, rural, and remote areas outside the two designated children's tertiary hospitals. The tertiary hospital paediatric CNCs were excluded as they are specialists in their clinical area and were not members of the NSW CHN paediatric CNC group which the research team was invited to attend. The researchers conducted a two-hour workshop on setting research priorities using a NGT. A total of sixteen CNCs attended the meeting and their demographic characteristics were collected (see Table 1).

### 3.4. Data Collection and Analysis

**Figure 1:**
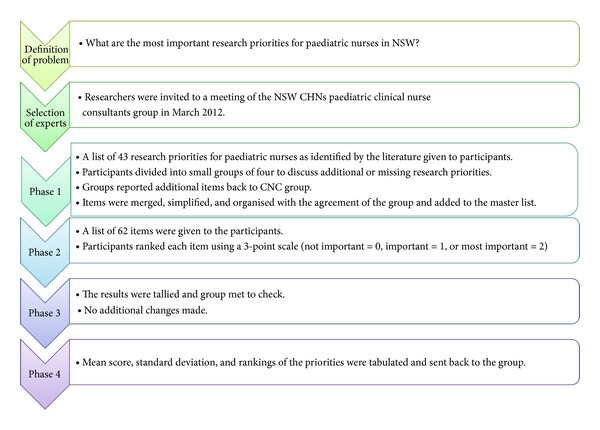
Nominal group technique process used in this study.

A workshop was conducted using a nominal group technique (NGT) during a meeting of the NSW CHN Paediatric CNC group on 21st March 2012. The meeting participants were asked to think about the research question: *What are the most important research priorities for paediatric nurses in NSW? *The NGT was conducted in four phases (see Figure 1).

**Phase 1**

The researchers gave a forty-three item list of research topics for paediatric nurses identified from the literature to the NGT [15, 35]. This list was derived from a combination of the resulting top research priorities for paediatric nurses as identified by Moreno-Casbas et al., [35] and Wilson et al [15]. Fifteen items came from the Moreno-Casbas et al. [35] and 28 items came from Wilson et al. [15]. Each research topic was randomly assigned a number between 1 and 43 to determine their order on the combined list used in this study.

The participants were divided into small groups of four and asked to read the list and add any missing or additional items. A forty minute period was allowed for them to discuss the pros and cons of each item including their additions. The small groups then came back together, and using a round robin, each participant in turn stated one or more additional items. The items were written on a white board and discussion by the group was limited to clarification only. Using a laptop, the additional items listed on the board were merged, simplified and organised with the agreement of the group (removing any overlap and duplication). The additional items were added to a master list containing the original 43 items.

**Phase 2**

A new typed list of research topics was provided to each participant and they were asked to rank each item using the point scale provided (Not Important (N/A) = 0, Important = 1 or Most Importance = 2). The researchers then tallied the responses.

**Phase 3**

The results of Phase 2 were provided to the participants at the meeting and a final consensus of agreement was achieved. Before leaving the meeting, the participants were asked to complete a demographic survey with questions on gender, age, position title and highest paediatric/academic qualifications. The advantage of the method was that the initial data analysis was achieved at the same time as data collection and the participants had a sense of ownership in the results. Following the meeting the responses were entered into Excel and further analysed. This included calculation of means and standard deviations and tabulation of results.

**Phase 4**

The mentor revisited the NSW CHNs Paediatric CNC group at their next meeting in May 2012 to go over the findings from the NGT and to revisit the priorities with the group. The group made no changes to their original priorities. The mentor explored with the members the research methods that could be utilised to commence research projects based on the NGT findings.

### 3.5. Ethics

The University of Western Sydney Human Research Ethic Committee was consulted and advised that according to the National Health Research Medical Council guidelines [42] formal ethics approval was not required. The committee advised that the researchers needed to have their attendance and activities recorded in the meeting minutes as the CNC group invited the researchers to conduct the research priority setting exercise. The workshop was listed on the meeting agenda and all paediatric nurses attending the meeting consented to participate in the workshop. The results from the nominal group discussion were recorded in the meeting minutes, and a copy was provided to the researchers with permission to publish the results. 

## 4. Results

### 4.1. The Participants

Sixteen generalist paediatric CNCs were members of the NSW CHN paediatric CNC group. Seven (46%) CNCs had a hospital certificate, four (25%) had a bachelor of nursing, and one (6.3%) had a bachelor of nursing honours degree, as an initial nursing qualification. The participants ranged in age from 30 to 58 years, with a mean age of 44 years. Fourteen of the CNCs had a paediatric qualification, with nine (56.3%) at masters level and five (31.3%) at graduate certificate level.

### 4.2.  Phase 1

Nineteen research priorities were added by the CNCs to the original master list of 43 items.

### 4.3. Phase 2/Phase 3

Table 2 displays the top 30 research topics on the master list (62 items) which had a participant mean of 1 (important) or greater. They are listed in descending order of importance to the group according to the mean score. Included in this list were 15 (50%) of the additional research topics added by the NGT participants. Of the 30 research topics, five items (11, 46, 47, 15, and 60) were related to pain either its assessment or effectiveness of actions to manage it. Item 11, encompassing a broad view of issues related to pain, was ranked as the top research priority. Four items (55, 56, 59, and 32) were related to issues in the Emergency Department (ED). Three of these were added by the participants to the master list and included reasons why children re-present to ED (55), parent expectations of the ED (56), and when observations are done in ED (59)? Item 32, explore families' reasons for presenting to the Emergency Department, came from the Wilson et al. [15]. 

To determine the focus of the priority, the research topics were grouped by the researchers using Wilson et al.'s [15] categories: (1) research topics of greatest value to patients, (2) research topics of greatest value to the families, and (3) research topics that would most facilitate health in children and young people and reduced hospitalisation (see Table 2). The grouped items presented in Table 2 were emailed to the CNC group and confirmed at their next meeting.

1. Fifteen priorities have greatest value for the patient (i.e., clinical issues, psychosocial issues, safety issues, quality care, and role competence issues), the top three issues ranked by the NGT were 11, 46, and 47, and they all related to pain.
2. Two priorities have the greatest value for the family. These were parent expectations of the ED and paediatric units (56) and assessed parent understanding and usefulness of information provided (printed and other modes) regarding child's care and discharge, including long-term outcomes (22).
3. Twelve priorities would most facilitate the health of the children and reduce hospital admission (clinical issues, health education issues, and models of care). The top three ranked by the nurses were the reasons children re-present at ED (55), non-compliance of clinical practice guidelines (CPG) by GPs and visiting medical officers (53), and exploring the families' reasons for presenting to ED (32).

### 4.4.  Phase 4

At the next meeting of the NSW CHNs paediatric CNC group in May 2012 the findings from the NGT were discussed and further refined. Research methods that could be utilised to commence research projects based on the NGT findings were discussed, including conducting systematic reviews of the literature. The CNCs decided to pursue the two priorities: reasons for children re-presenting to ED and a comparison of the use of high-flow and low-flow nasal prongs in children with bronchiolitis. While pain-related topics were the top priorities, the group felt they wanted to pursue the two nominated topics as they were currently an issue in their fields of practice. Another priority highlighted was medical clinician adherence to clinical standard guidelines, however, this was already being pursued by one of the CNCs in a local multidisciplinary project. The group established two subgroups of four to five nurses to conduct systematic reviews of the literature on both topics. The mentor was available by phone or face-to-face meetings which were followed up by one of the subgroups. Eight months after the research priority setting exercise, group one continued with their literature review on readmission to emergency, while group two, examining high and low oxygen, prepared an ethics application under the guidance of the mentor. 

## 5. Discussion

The priorities set by the CNC group reflect results from other studies [15, 35] with pain management being the top priority research area. Ongoing poor management of pain continues to be an issue [43]. The top priority identified by Wilson et al. [15] was related to medication errors but was ranked at 10 by the nurses in the current study, therefore, reflecting a need to examine research in this area. It is clear that the research priorities considered of greatest value to improving practice were those related to patient care and reduction of readmissions to hospital according to Wilson et al. [15] classification. This is similar to past findings [15, 32]. One issue not addressed in this process of ranking research needs in paediatrics is what consumers need or want. As suggested by Gillies [44], multistakeholder groups including the children, parents, and practitioners need to be used in setting research priorities. 

This project has started the journey to engage CNCs in the research process and to establish relationships which can enable their further development in the skills and knowledge required for research in paediatric nursing. The group has worked together with a common focus, thus, the beginnings of a research culture have been established. In establishing these groups of nurse researchers, the nurses can become motivated and supported in fulfilling the research domain of their CNC role. 

Kajermo et al. [45] argue that CNCs are ideally placed to promote research-based nursing practice if prepared and supported. CNCs, however, need support and funds from health services management to achieve this. As suggested by Corchon et al. [46] it could be useful to have management involved with specialist nurses in setting research priorities. This will integrate management and clinical priorities and may assist the motivation for management to support clinical research The CNCs need to be educated to the masters level and as seen in our sample only 56.3%. (*n* = 9) had completed education to this level, although it is recommended by NSW Health [7]. Management needs to find ways to support the CNCs, in relation to time release and financial support, for example, paid study leave. While in-house research education continues in hospitals across NSW, when a speciality is so small it makes it difficult for specialities to collaborate.

Working together on the systematic review and establishing collaborative projects across organisational and geographical boundaries, with ongoing mentoring, will help develop and establish this research culture. As suggested by Bishop and Freshwater [12] participating in a research culture should become a part of every practitioner's role. However, the research culture will not flourish if the organisations where the nurses are employed do not value research and provide an environment where it is encouraged [47, 48].

## 6. Implications for Nursing Practice

As indicated in the findings of this study the CNCs held similar priorities as paediatric nurses in other studies. However, the process has opened new paths to mentor these CNCs and develop research collaborations across organisational and geographical boundaries. As shown by Gagliardi et al. [49], mentoring can facilitate the development of research capacity in nurses and encourage knowledge transfer into clinical practice. This capacity is stressed in the study by Mannix et al. [50] as necessary for nurses to become clinical scholars which should be the aim of CNCs. 

Leadership development is essential for CNCs as they appear to not address this domain, as well as they could [1, 6]. Initiating knowledge transfer to practice and especially to policy development in order to enhance patient care is one bridge to this development. However, resources and personal support from management are essential for any change to occur [49].

## 7. Limitations of the Study

A limitation of this study was that only one group of expert Paediatric nurses was used for the NGT, however, 16 is an ideal number of participants for an NGT. This constituted 69.5% of the total population of generalist paediatric CNCs in NSW. The benefit of this one-off process is that a substantial amount of information can be gathered in a relatively short time [39]. It also allowed research relationships to develop. 

## 8. Conclusion and Recommendation

This study has opened the way for a group of CNCs in paediatric nursing to combine their research capacity and influence clinical knowledge. As recommended by McCance et al. [51] the first step in research capacity building is encouraging nurses to value research and legitimise it as an essential activity to improve their professional practice. Further work is required to involve consumers of health care for children. Repeating the research with a group of consumers should provide valuable information for clinicians. It could be useful to survey CNCs in a tertiary paediatric hospital in NSW using the research priorities developed in this study. This study provides a model others can use to encourage research culture development in groups of CNCs. 

## Figures and Tables

**Table 1:** Demographic characteristics of participants in NGT (*n* = 16).

Demographic characteristic	*n*	%
Gender		
Male	2	12.5
Female	14	87.5
Age in years		
Range	30–58	
Mean	44	
30–40	4	25.0
41–50	8	50.0
51–60	3	18.8
Missing	1	6.3
Position title		
Paediatric CNC	16	100.0
Highest qualification in paediatrics		
BA honours paediatric nursing	1	6.3
Graduate certificate	5	31.3
Masters	9	56.3
Missing	1	6.3
First nursing qualification		
BA Honours paediatric nursing	1	6.3
Bachelor of nursing	4	25.0
Diploma of applied science nursing	2	12.5
Enrolled nurse	1	6.3
Hospital certificate (RN)	7	43.8
Missing	1	6.3

**Table 2:** Top research priorities nominated by the participants from the combined master list from literature and those they nominated (italicised items from literature [15, 35]).

Research topic number	Research topic		Mean	SD	Rank	Research category
11	Determine how pain assessment impacts on pain management (including nurses' perceptions of pain assessment, effectiveness of different analgesic groups, and postoperative pain management)	W	1.75	0.45	1	Patient
46	Why paediatric nurses do not use pain scores for children?	G	1.56	0.51	3	Patient
47	Does a nurse-initiated pain assessment lead to better pain management?	G	1.56	0.51	3	Patient
55	Reasons for children representing to the emergency department	G	1.56	0.63	3	Reduce hospitalisation
53	Non-compliance of clinical practice guidelines by doctors?	G	1.5	0.63	5	Reduce hospitalisation
54	A comparison of the use of high-flow and low-flow nasal prongs in children with bronchiolitis	G	1.44	0.63	7	Patient
56	What are parent expectations of nursing care in emergency departments and paediatric units?	G	1.44	0.73	7	Family
59	How frequently are observations preformed on paediatric patients in Emergency Departments?	G	1.44	0.73	7	Patient
12	Evaluate effect of critical incidents feedback on subsequent occurrence of critical incidents	W	1.38	0.62	10	Patient
37	Identify strategies to reduce medication errors	W	1.38	0.72	10	Patient
52	Should there be any difference between nurse ratios and acuity for paediatrics and adult patients?	G	1.38	0.62	10	Patient
32	Explore families' reasons for presenting to the emergency department	W	1.31	0.70	13	Reduce hospitalisation
44	Do foster kids differ from nonfoster kids when going home with a chronic respiratory condition? Does the hospital in the home work for foster kids?	G	1.31	0.79	13	Reduce hospitalisation
62	Reasons why there is a delay in contacting NETS when a child needs retrieval	G	1.31	0.79	13	Patient
14	Identify the nurse-practitioner role in paediatrics to improve care delivery and outcomes	W	1.25	0.45	15.5	Patient
51	Impact of technology on bedside care	G	1.25	0.68	15.5	Patient
13	Explore the impact of parental involvement in hospital care including decision-making (impact on child, parent, and staff)	W	1.19	0.66	19	Patient
26	Explore models of ambulatory care/hospital in the home/community services to assist in care of children with chronic/complex care needs	W	1.19	0.66	19	Reduce hospitalisation
36	Identify reasons for parental noncompliance of treatment and explore strategies to increase compliance (e.g., asthma prevention and management and children with psychiatric disorders)	*W *	*1.19 *	0.54	19	Patient
58	Skill and knowledge retention following paediatric resuscitation education programs for health professionals	G	1.19	0.66	19	Reduce hospitalisation
61	How do paediatric nurses interpret a paediatric AVPU score?	G	1.19	0.66	19	Patient
1	Identify where nurse practitioners can be employed within children's health care	W	1.13	0.50	23	Reduce hospitalisation
18	Investigate effects of therapeutic play/distraction on children's anxiety and outcomes in hospital (effects on clinical holding)	W	1.13	0.81	23	Patient
45	Identify the needs of rural paediatrics in NSW	G	1.13	0.62	23	Reduce hospitalisation
15	Explore the impact of pain and anxiety on children who regularly require surgery	W	1.06	0.44	26.5	Patient
22	Assess parent understanding and usefulness of information provided (printed and other modes) regarding child's care and discharge, including long-term outcomes	W	1.06	0.68	26.5	Family
31	Examine practices, community treatments and prevention of common causes of childhood hospitalisation (e.g., otitis media, dental caries).	W	1.06	0.57	26.5	Reduce hospitalisation
34	Investigate the impact of nurse led care in acute care settings.	W	1.06	0.77	26.5	Reduce hospitalisation
2	To analyse the co-ordination between hospitals and primary care settings for the continuity of nursing care	M	1	0.73	29.5	Reduce hospitalisation
60	The role of intranasal fentanyl in post-operative tonsillectomy pain management	G	1	0.52	29.5	Patient

Key: W: Wilson et al. 2010 [15], M: Moreno-Casbas et al. 2001 [35], G: Paediatric nurse group.
